# Implications of Older Adult Attitudes Toward the Preventability of Falls for Health Promotion

**DOI:** 10.1093/geroni/igaa057.2797

**Published:** 2020-12-16

**Authors:** Gwen Bergen, Janice Mark, Ankita Henry

**Affiliations:** Centers for Disease Control and Prevention, Atlanta, Georgia, United States

## Abstract

Older adults’ behavioral stage of change for adopting fall prevention interventions, and their use of evidence-based interventions are not well understood. A survey was administered to older adults (65 years+) (n=1063) to understand their stage of change and fall prevention behaviors. Descriptive statistics were calculated and logistic regression conducted to determine factors most related to stage. The distribution of subjects by stage was precontemplation (17%), contemplation (2%), preparation (5%), action (15%), and maintenance (61%). The strongest variable related to being in an action stage (preparation, action, maintenance) was screening positively for fall risk (Risk Ratio: 8.7, 95% CI: 5.4, 14.1). The most common preventive actions for those in an action stage were taking Vitamin D (37%), and having vision tested (30%). Older adults at risk for a fall are ready to take action to prevent falls; health promotion should focus on increasing knowledge and use of different evidence-based interventions.

